# World Trade Center Health Program — United States, 2012−2020

**DOI:** 10.15585/mmwr.ss7004a1

**Published:** 2021-09-10

**Authors:** Alejandro Azofeifa, Gayatri R. Martin, Albeliz Santiago-Colón, Dori B. Reissman, John Howard

**Affiliations:** ^1^World Trade Center Health Program, National Institute for Occupational Safety and Health, CDC; ^2^National Institute for Occupational Safety and Health, CDC

## Abstract

**Problem/Condition:**

After the September 11, 2001, terrorist attacks on the United States, approximately 400,000 persons were exposed to toxic contaminants and other factors that increased their risk for certain physical and mental health conditions. Shortly thereafter, both federal and nonfederal funds were provided to support various postdisaster activities, including medical monitoring and treatment. In 2011, as authorized by the James Zadroga 9/11 Health and Compensation Act of 2010, the CDC World Trade Center (WTC) Health Program began providing medical screening, monitoring, and treatment of 9/11-related health conditions for WTC responders (i.e., persons who were involved in rescue, response, recovery, cleanup, and related support activities after the September 11, 2001, terrorist attacks) and affected WTC survivors (i.e., persons who were present in the dust or dust cloud on 9/11 or who worked, lived, or attended school, child care centers, or adult day care centers in the New York City disaster area).

**Reporting Period Covered:**

2012–2020.

**Description of System:**

The U.S. Department of Health and Human Services WTC Health Program is administered by the director of CDC’s National Institute for Occupational Safety and Health. The WTC Health Program uses a multilayer administrative claims system to process members’ authorized program health benefits. Administrative claims data are primarily generated by clinical providers in New York and New Jersey at the Clinical Centers of Excellence and outside those states by clinical providers in the Nationwide Provider Network. This report describes WTC Health Program trends for selected indicators during 2012–2020.

**Results:**

In 2020, a total of 104,223 members were enrolled in the WTC Health Program, of which 73.4% (n = 76,543) were responders and 26.6% (n = 27,680) were survivors. WTC Health Program members are predominantly male (78.5%). The median age of members was 51 years (interquartile range [IQR]: 44−57) in 2012 and 59 years (IQR: 52−66) in 2020. During 2012−2020, enrollment and number of certifications of WTC-related health conditions increased among members, with the greatest changes observed among survivors. Overall, at enrollment, most WTC Health Program members lived in New York (71.7%), New Jersey (9.3%), and Florida (5.7%). In 2020, the total numbers of cancer and noncancer WTC-related certifications among members were 20,612 and 50,611, respectively. Skin cancer, male genital system cancers, and in situ neoplasms (e.g., skin and breast) are the most common WTC-related certified cancer conditions. The most commonly certified noncancer conditions are in the aerodigestive and mental health categories. The average number of WTC-related certified conditions per certified member is 2.7. In 2020, a total of 40,666 WTC Health Program members received annual monitoring and screening examinations (with an annual average per calendar year of 35,245). In 2020, the total number of WTC Health Program members who received treatment was 41,387 (with an annual average per calendar year of 32,458).

**Interpretation:**

Since 2011, the WTC Health Program has provided health care for a limited number of 9/11-related health conditions both for responders and survivors of the terrorist attacks. Over the study period, program enrollment and WTC certification increased, particularly among survivors. As the members age, increased use of health services and costs within the WTC Health Program are expected; chronic diseases, comorbidities, and other health-related conditions unrelated to WTC exposures are more common in older populations, which might complicate the clinical management of WTC-related health conditions.

**Public Health Action:**

Analysis of administrative claims data in the context of WTC research findings can better clarify the health care use patterns of WTC Health Program members. This information guides programmatic decision-making and might also help guide future disaster preparedness and response health care efforts. Strengthening the WTC Health Program health informatics infrastructure is warranted for timely programmatic and research decision-making.

## Introduction

On the morning of September 11, 2001, four U.S. commercial airplanes, full with fuel for transcontinental flights, were hijacked (*1*). Two of these airplanes crashed into the two towers of the World Trade Center (WTC) complex in lower Manhattan in New York City (NYC), New York (*1*). The third and fourth airplanes crashed into the Pentagon in Arlington, Virginia, and into a field near Shanksville, Pennsylvania, respectively (*1*). Both airplanes in NYC exploded on impact, and intense heat from burning jet fuel caused both towers to collapse, resulting in nearly 2,800 deaths and thousands more injuries (*1*,*2*). Soon after, the third airplane crashed into the west wall of the Pentagon, the headquarters of the U.S. Department of Defense, resulting in 125 deaths and approximately 50 injured persons who were treated at local hospitals (*3*). The fourth airplane crashed into a field near Shanksville, Pennsylvania, after passengers and crew members intervened to prevent the completion of an additional targeted terrorist attack (*1*). All passengers and crew members of the four flights died at the crash sites.

As a result of these attacks, thousands of persons at the crash sites and nearby areas were exposed to numerous environmental toxins and hazardous materials because of a massive outdoor cloud of dust and debris, indoor dust, and fumes from fires (*4*). Shortly after, thousands joined the rescue, recovery, and cleanup efforts at the four crash sites (*5*). As a result, approximately 400,000 persons were determined to be at increased risk for adverse physical and psychological health effects (*4*,*6*). Initial response efforts (e.g., triage and treatment of injured persons, surveillance, and clinical and environmental assessment activities) were conducted by local public health authorities (*3*,*5*,*7*).

The New York City Department of Health and Mental Hygiene activated emergency protocols and its emergency operations center and then collaborated with the Agency for Toxic Substances and Disease Registry to develop the WTC Health Registry (*5*). This registry was created to evaluate short-term and long-term physical and mental health effects among persons exposed to the WTC disaster and its aftermath and is the largest postdisaster environmental health registry in the United States (*8*). Five survey waves have been conducted (2003–2004, 2006–2008, 2011–2012, 2015–2016, and 2020–2021); WTC Health Registry enrollment was open during September 2003–November 2004 and is no longer open to new registrants (*8*). Soon thereafter, additional federal and nonfederal funding was provided to support various local postdisaster activities, including medical monitoring and treatment. Some of these health care–related activities were conducted by local hospitals and clinics through cooperative agreements and grants. During the first decade (2001−2011) after the terrorist attacks, the health monitoring and treatment provided to affected persons was performed by the following programs: the Medical Monitoring and Treatment Program, the National Responder Program, and the WTC Environmental Health Center Community Program (*9*) (Table 1). In addition to local efforts to monitor short-term and long-term physical and mental health effects (WTC Health Registry) and provide earlier medical monitoring and treatment, the U.S. Department of Justice provided initial financial compensation to any person (or relative of a deceased person) who was physically injured or killed as a result of the September 11, 2001, terrorist attacks, by establishing the September 11th Victim Compensation Fund (VCF).

**TABLE 1:** Description of 9/11-related health programs, including early health monitoring and treatment programs, the World Trade Center Health Program, the September 11th Victim Compensation Fund, and the World Trade Center Health Registry

Program	Responsible entity	Description
**Early monitoring and treatment programs established before 2011**		
Medical Monitoring and Treatment Program* (responders)	New York City (NYC) Clinical Centers of Excellence (Mount Sinai School of Medicine)	Soon after the September 11, 2001, terrorist attacks, a group of experts and physicians from the Mount Sinai Irving J. Selikoff Center for Occupational and Environmental Medicine developed a medical screening program to evaluate the health status of World Trade Center (WTC) responders (persons who were involved in rescue, response, recovery, cleanup, and related support activities after the September 11, 2001, terrorist attacks).^†^ Early in 2002, federal funding was committed to establish the WTC Worker and Volunteer Medical Screening Program. In 2004, in addition to the initial health screenings, the program was expanded to provide periodic monitoring examinations. Later that year, through various funding mechanisms, the WTC Health Effects Treatment Program was established. In 2006, federal funding was provided for physical and mental health diagnostic and treatment services to the WTC Medical Monitoring Program (later renamed the WTC Medical Monitoring and Treatment Program). The program medical providers were individually funded as Clinical Centers of Excellence and included Mt. Sinai Medical Center, New York University School of Medicine—Bellevue Hospital Center, Queens College, Richmond University Medical Center, State University of New York—Stony Brook, and University of Medicine and Dentistry of New Jersey (Mount Sinai Consortium). Simultaneously in those years, the FDNY responders (firefighters, fire officers, and the FDNY and emergency medical services [EMS] workers) rapidly implemented a monitoring and treatment system through the department’s Bureau of Health Services. In December 2001, through federal funding, this medical monitoring and treatment program included FDNY volunteers and FDNY EMS retirees (additional information available at https://www.mountsinai.org/care/occupational-health/services-programs/wtc; https://www.fdnywtcprogram.org).
	Fire Department of the City of New York (FDNY)	
National Responder Program (responders)*	Mount Sinai (2006−2008)	In 2006, federal funding was provided for physical and mental health diagnostic and treatment services to the WTC responders that live outside of the New York metropolitan area through a network of providers via a contract between Mount Sinai and a third party. In 2008, Logistics Health, Inc., was awarded the contract for the National Responder Program (additional information available at https://logisticshealth.com/index.aspx).
	Logistics Health, Inc. (2008 through July 1, 2011) (health providers nationwide)	
World Trade Center Environmental Health Center Community Program (survivors)*	New York City Clinical Center of Excellence (NYC Health and Hospitals Corporation)	In 2008, federal funds were appropriated to provide screening, diagnostic, and treatment services to residents, students, and others who were affected by the September 11, 2001, terrorist attacks through the WTC Environmental Health Center Community Program, operated under a CDC-sponsored grant to the NYC Health and Hospitals Corporation (additional information available at https://www.nychealthandhospitals.org/services/wtc-environmental-health-center).
**Programs established, reopened, or funded by the Zadroga Act**^§^		
World Trade Center Health Program (responders and survivors)	U.S. Department of Health and Human Services, CDC, National Institute for Occupational Safety and Health (NIOSH)	Established in 2011 by the James Zadroga 9/11 Health and Compensation Act of 2010 (Zadroga Act), the WTC Health Program was created to replace the earlier 9/11-related programs and began providing medical screening, monitoring, and treatment of 9/11-related health conditions for affected responders and survivors. (A responder is defined as a person who was involved in rescue, response, recovery, cleanup, and related support activities after the September 11, 2001, terrorist attacks; a survivor is defined as a person who was present in the dust or dust cloud on 9/11 or who worked, lived, or attended school, child care centers, or adult day care centers in the NYC disaster area.) In addition, the Zadroga Act directed the WTC Health Program to conduct and coordinate education and outreach, collect and analyze physical and mental health data, and research health conditions resulting from the terrorist attacks. Responders and survivors are the two primary groups who are eligible to enroll in WTC Health Program. In 2015, the WTC Health Program was reauthorized for 75 years,^¶^ providing federal funding through 2090 (additional information available at https://www.cdc.gov/wtc/).
September 11th Victim Compensation Fund (VCF)	U.S. Department of Justice	VCF provides financial compensation for physical injury, illness, or death due to qualifying circumstances and health conditions arising from the September 11, 2001, terrorist attacks. In 2001, the U.S. Congress established the original VCF (VCF1) in an effort to provide financial compensation to any person (or relative of a deceased person) who was physically injured or killed as a result of the attacks. The original VCF1 closed in 2004.** In 2011, under the Zadroga Act, VCF was reopened and authorized to operate.^††^ In 2015, VCF was reauthorized, extending claim filing deadlines and increasing funding.^§§^ In 2019, VCF received permanent authorization, extending deadlines and appropriating funds that might be needed to pay all approved claims.^§§^ VCF serves eligible responders and survivors associated with the NYC disaster area and qualifying responders to the other 9/11 terrorist attacks in Arlington, Virginia, and Shanksville, Pennsylvania. Families of deceased persons who qualify for VCF funds may apply to VCF on the decedent’s behalf. In general, VCF requires that claimants be enrolled in and have their physical conditions certified for treatment by the WTC Health Program to process a VCF claim and award compensation. Otherwise, the WTC Health Program and the VCF administer their programs independently (additional information available at https://www.vcf.gov/).
World Trade Center Health Registry	New York City Department of Health and Mental Hygiene	The WTC Health Registry was established in 2002 by New York City Department of Health and Mental Hygiene and the federal ATSDR to evaluate short-term and long-term physical and mental health effects from the September 11, 2001, terrorist attacks and the aftermath. Since its inception, WTC Health Registry funding has been provided by local or federal agencies (Federal Emergency Management Agency). In 2009, administration of the WTC Health Registry was transferred from ATSDR to NIOSH. In 2011, funding became more stable after enactment of the James Zadroga 9/11 Health and Compensation Act of 2010 (Zadroga Act). Enrollment was open during September 2003–November 2004 and is no longer open to new registrants. The WTC Health Registry collects information about the physical and mental health conditions of enrollees (i.e., rescue and recovery personnel, cleanup workers, residents, students and school staff members, and building occupants and bystanders in lower Manhattan) during a 30-minute telephone or in-person interview questionnaire. WTC Health Registry surveys have been administered in five survey waves (2003–2004, 2006–2008, 2011–2012, 2015–2016, and 2020–2021). Original, follow-up, and new survey questions cover a wide range of topics on the different survey waves such as location during 9/11 and level of exposure to dust, smoke, and debris; follow-up questions for specific groups; common health conditions and symptoms; medications and hospitalizations; mental health treatment and hospitalizations; asthma; time course of posttraumatic stress disorder; serious psychological distress; depression and anxiety; cancer screening and cancer family history; and mental health treatment. Participants in the WTC Health Registry are not necessarily automatically enrolled in the WTC Health Program and VCF (additional information available at https://www1.nyc.gov/site/911health/index.page).
	Agency for Toxic Substances and Disease Registry (ATSDR)	

* Responders and survivors who were enrolled in the federally sponsored predecessor programs (i.e., responders and survivors who were enrolled the Medical Monitoring and Treatment Program, National Responder Program, or the WTC Environmental Health Center Community Program) before July 1, 2011, were automatically enrolled in the WTC Health Program. These earlier monitoring and treatment programs medical providers still exist by name (now known as Clinical Centers of Excellence), function, and location but now are funded by federal law (Zadroga Act). A list of current WTC Health Program Clinical Centers of Excellence is available at https://www.cdc.gov/wtc/clinics.html.

^† ^Moline JM, Herbert R, Levin S, et al. WTC medical monitoring and treatment program: comprehensive health care response in aftermath of disaster. Mt Sinai J Med 2008;75:67–75.

^§^ James Zadroga 9/11 Health and Compensation Act of 2010 (Zadroga Act). Pub. L. 111-347, 124 Stat. 3623 (Jan. 2, 2011). https://www.govinfo.gov/content/pkg/PLAW-111publ347/pdf/PLAW-111publ347.pdf

^¶^ James Zadroga 9/11 Health and Compensation Reauthorization Act, 2016. Pub. L. 114-133, 129 Stat. 2996 (Dec. 18, 2015). https://www.govinfo.gov/content/pkg/PLAW-114publ113/pdf/PLAW-114publ113.pdf

** September 11th Victim Compensation Fund of 2001. Pub. L. 107–42, 115 Stat. 237, Title IV—Victim Compensation) (Sept. 22, 2001). https://www.govinfo.gov/content/pkg/PLAW-107publ42/pdf/PLAW-107publ42.pdf

^††^ U.S. Department of Justice. James Zadroga 9/11 Health and Compensation Act of 2010. Fed Regist 2011 Aug 31;76(169):54112. https://www.govinfo.gov/content/pkg/FR-2011-08-31/pdf/2011-22295.pdf

^§§^ U.S. Department of Justice. James Zadroga 9/11 Victim Compensation Fund Reauthorization Act. Fed Regist 2016 June 15;81(115):38936–48. https://www.vcf.gov/sites/vcf/files/resources/HR2029-759.pdf; Never Forget the Heroes: James Zadroga, Ray Pfeifer, and Luis Alvarez Permanent Authorization of the September 11th Victim Compensation Fund Act. Pub. L. 116–34, 133 Stat. 1040 (July 29, 2019). https://www.congress.gov/116/plaws/publ34/PLAW-116publ34.pdf

On July 1, 2011, the CDC World Trade Center Health Program was created under the James Zadroga 9/11 Health and Compensation Act of 2010 (Zadroga Act) to continue and expand previous 9/11-related programs and began providing medical screening, monitoring, and treatment of a limited number of specific 9/11-related health conditions for affected members, categorized as responders or survivors (*10*). A responder is defined as a person who was involved in rescue, response, recovery, cleanup, and related support activities after the September 11, 2001, terrorist attacks; a survivor is defined as a person who was present in the dust or dust cloud on 9/11 or who worked, lived, or attended school, child care centers, or adult day care centers in the NYC disaster area. The Zadroga Act also authorized ongoing research on the physical and mental health of affected persons and the maintenance of the WTC Health Registry. In addition, the Zadroga Act reauthorized and extended VCF claim filing deadlines. This report describes WTC Health Program trends for selected indicators during 2012–2020 and provides insights into the development and evolution of a national postdisaster health program. The findings can be used to assess the quality, relevance, and timeliness of WTC Health Program administrative and research data to effectively monitor 9/11-related health care and to guide future disaster preparedness and response health efforts.

## Methods

### James Zadroga 9/11 Health and Compensation Act of 2010

On January 2, 2011, the Zadroga Act was signed into law by President Barack H. Obama (*10*). This law established the WTC Health Program, a limited health benefits plan for enrolled members affected by the September 11, 2001, terrorist attacks (Table 1). The WTC Health Program is not intended to replace full general health insurance coverage for all health care needs. The program is administered by the director of CDC’s National Institute for Occupational Safety and Health, a part of the U.S. Department of Health and Human Services (*11*). On July 1, 2011, the WTC Health Program began providing the following no-cost health benefits to enrolled members: 1) medical monitoring and treatment benefits for responders, defined as emergency responders and recovery and cleanup workers who responded to the September 11, 2001, terrorist attacks in NYC, on the Pentagon, and in Shanksville, Pennsylvania, and 2) initial health evaluation (screening), monitoring, and treatment benefits for survivors, defined as persons who were present in the dust or dust cloud on 9/11 or who worked, lived, or attended school, child care centers, or adult day care centers in the NYC disaster area (*10*). In addition to providing medical screening, monitoring, and treatment for 9/11-related health conditions, the WTC Health Program is mandated to conduct education and outreach, collect and analyze physical and mental health data, and research health conditions resulting from the terrorist attacks. More detailed information about the Zadroga Act and the WTC Health Program is available (*10*,*11*) (Box 1).

BOX 1 **History of the World Trade Center Health Program**

**September 11, 2001**

- **8:46 a.m.** American Airlines flight 11 crashed into the north tower of the World Trade Center (WTC) complex in New York City (NYC), New York.
- **9:03 a.m.** United Airlines flight 175 crashed into the south tower of the WTC complex in NYC.
- **9:37 a.m.** American Airlines flight 77 crashed into the west wall of the Pentagon, the headquarters of the U.S. Department of Defense, in Arlington, Virginia.
- **10:03 a.m.** United Airlines flight 93 crashed into a field near Shanksville, Pennsylvania.
- Immediate emergency response initiated by local health and federal authorities.*

**2002**

- Health screening examinations began for workers involved with response, recovery, or cleanup operations at the WTC disaster sites. The examinations were funded through contracts awarded by CDC’s National Institute for Occupational Safety and Health (NIOSH) to the Mount Sinai School of Medicine (Mount Sinai) and by CDC’s National Center for Environmental Health to the Fire Department of the City of New York (FDNY). Mount Sinai subcontracted with specialized occupational health clinics in the New York metropolitan area. Together, Mount Sinai and the other health clinics were collectively called the Mount Sinai Consortium. Funding was provided through emergency, discretionary appropriations.
- The WTC Health Registry was established by the NYC Department of Health and Mental Hygiene and administered by the Agency for Toxic Substances and Disease Registry (ATSDR).^†^

**2004**

- Initial health screenings were expanded to add periodic monitoring examinations. Clinics providing screening and monitoring examinations were funded through cooperative agreements awarded by NIOSH to Mount Sinai (for the Mount Sinai Consortium) and FDNY and collectively called the WTC Medical Monitoring and Treatment Program.

**2006**

- Diagnostic and treatment services were added to provide medical care for WTC-related health conditions. Health monitoring and treatment to affected persons were coordinated and performed by the Mount Sinai Consortium and FDNY, collectively called the WTC Medical Monitoring and Treatment Program.
- Diagnostic and treatment services outside the New York metropolitan area were provided by a network of medical providers (i.e., the National Responder Program). Funding was provided through a direct contract between Mount Sinai and a third party.

**2008**

- Screening, diagnostic, and treatment services were expanded to residents, students, and others in NYC (survivors) by the WTC Environmental Health Center Community Program. Funding was provided through NIOSH’s sponsored grants to NYC Health and Hospitals Corporation (now referred to as NYC Health + Hospitals).
- NIOSH awarded the National Responder Program to Logistics Health, Inc.

**2009**

- Administration of the WTC Health Registry was transferred from ATSDR to NIOSH.

**2010**

- On December 22, the U.S. Congress passed the James Zadroga 9/11 Health and Compensation Act of 2010 (Zadroga Act).^§^

**2011**

- On January 2, President Barack H. Obama signed the Zadroga Act into law.
- The Zadroga Act established the WTC Health Program within NIOSH, which continued and expanded previous 9/11-related health programs (i.e., the Medical Monitoring and Treatment Program, the National Responder Program, the WTC Environmental Health Center Community Program, and the WTC Health Registry). The Zadroga Act also established the WTC Health Fund, to which annual appropriations are made through mandatory funding and which includes funds for a research component.
- On July 1, the WTC Health Program began operation.

**2012**

- Various types of cancers were added to the list of health conditions covered by the WTC Health Program.

**2015**

- On December 18, President Barack H. Obama signed the Consolidated Appropriations Act, 2016,^¶^ which reauthorized the WTC Health Program for 75 years, providing federal funding through 2090.

**2016**

- New-onset chronic obstructive pulmonary disease and acute traumatic injury were added to the list of health conditions covered by the WTC Health Program.
- A policy change by the U.S. Department of Justice’s September 11th Victim Compensation Fund (VFC) required VCF claimants to be certified by the WTC Health Program, resulting in a significant increase in the program’s enrollment numbers.

**2018**

- An additional clinic (William Street Clinic) was funded in the New York metropolitan area to provide monitoring and treatment services to support the large increase in enrollment of the survivor population.

**2019**

- On September 27, President Donald J. Trump signed the Continuing Appropriations Act, 2020, and Health Extenders Act of 2019,** which increased the number of new responders and new certified-eligible survivors the WTC Health Program could enroll by a total of 100,000 (50,000 additional responders and 50,000 additional certified-eligible survivors).

**2020**

- The WTC Health Program expanded telemedicine services to maintain medical services for members due to the impact of the COVID-19 pandemic.

* CDC. New York City Department of Health response to terrorist attack, September 11, 2001. MMWR Morb Mortal Wkly Rep 2001;50:821–2.

^†^ City of New York. NYC 9/11 Health; World Trade Center health registry. New York City, NY: City of New York; 2020. https://www1.nyc.gov/site/911health/index.page

^§^ James Zadroga 9/11 Health and Compensation Act of 2010 (Zadroga Act). Pub. L. 111-347, 124 Stat. 3623 (Jan. 2, 2011). https://www.govinfo.gov/content/pkg/PLAW-111publ347/pdf/PLAW-111publ347.pdf

^¶ ^James Zadroga 9/11 Health and Compensation Reauthorization Act, 2016. Pub. L. 114-133, 129 Stat. 2996 (Dec. 18, 2015). https://www.govinfo.gov/content/pkg/PLAW-114publ113/pdf/PLAW-114publ113.pdf

** Continuing Appropriations Act, 2020, and Health Extenders Act of 2019: Increasing Numerical Limitations of the World Trade Center Health Program. Pub. L. 116-59, 133 Stat. 1093, Sect. 1602 (Sept. 27, 2019). https://www.congress.gov/116/plaws/publ59/PLAW-116publ59.pdf

#### Eligibility, Enrollment, Initial Health Evaluation, and Annual Medical Monitoring

Responders and survivors are the two primary groups who are eligible for WTC Health Program enrollment. Responders include persons from the Fire Department of the City of New York (FDNY) (active or retired) and other specified responders (e.g., police, rescue, recovery, cleanup, and related support workers or volunteers) to the NYC disaster site; Pentagon responders; and Shanksville, Pennsylvania responders. Survivors include persons who were present in the dust or dust cloud on 9/11 or who worked, lived, or attended school, child care centers, or adult day care centers in the NYC disaster area. The Zadroga Act states that for survivors to be eligible for enrollment, they must report having symptoms that could be associated with a WTC-related health condition; this criterion is not required for responder eligibility. The criteria for survivor and responder eligibility are based on statutory and regulatory references of the Zadroga Act. In addition, to be eligible for enrollment in the WTC Health Program, responders and survivors must meet specified criteria related to exposure and potential for exposure (i.e., activity, location, time, and hours of exposure). Responder activities include services provided by workers affiliated with an employer or unpaid volunteer on-site work involving rescue, recovery, debris clean up, worker support services, or other related services.

**FIGURE 1:**
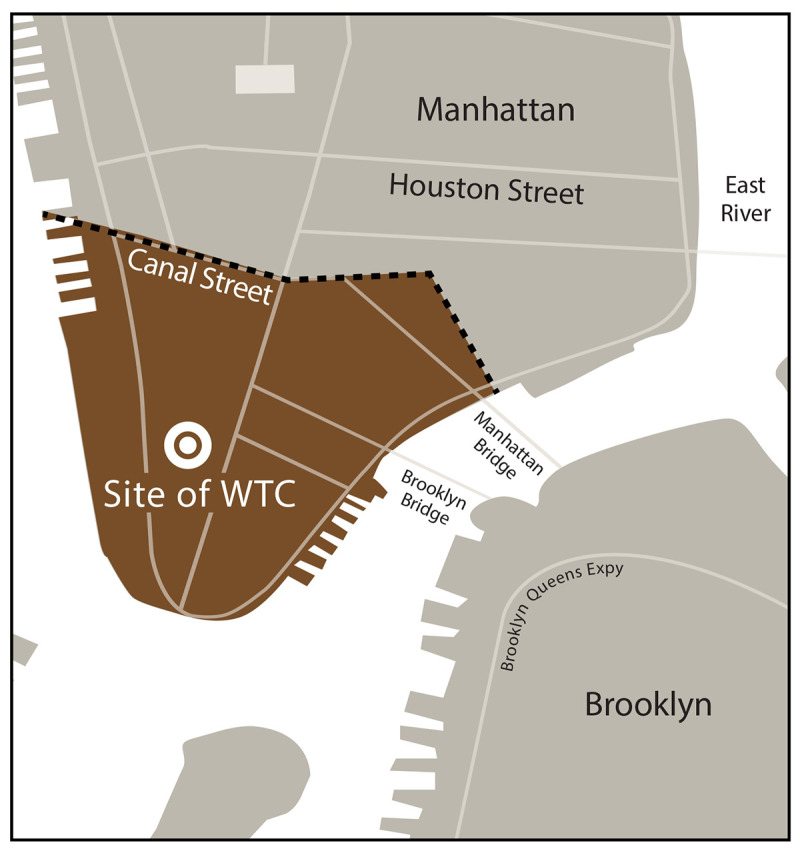
New York City response area* used to determine enrollment eligibility for responders^†^ to the 9/11 attacks — World Trade Center Health Program **Abbreviations:** NYC = New York City; WTC = World Trade Center. * The NYC response area for the WTC Health Program is defined as the area of lower Manhattan south of Canal Street. The area also includes WTC-related locations not shown, such as certain barge loading piers and the Staten Island Landfill. Some eligible responder duties might have been performed outside this area, such as vehicle maintenance. ^† ^Responder is defined as a person who was involved in rescue, response, recovery, clean-up, and related support activities after the September 11, 2001, terrorist attack.
  - Survivors: The NYC disaster area for survivor eligibility is defined as the area in Manhattan south of Houston Street and any block in Brooklyn wholly or partially contained within a 1.5-mile radius of the former WTC complex (Figure 2).

Location eligibility for responders and survivors is as follows:

1. NYC area site
  - Responders: The response area for responder eligibility is defined as the area in lower Manhattan south of Canal Street, including the former WTC complex (Figure 1), as well as the Port Authority Trans-Hudson Tunnel, the NYC chief medical examiner’s office, the Staten Island Landfill, and certain barge loading piers.

**FIGURE 2:**
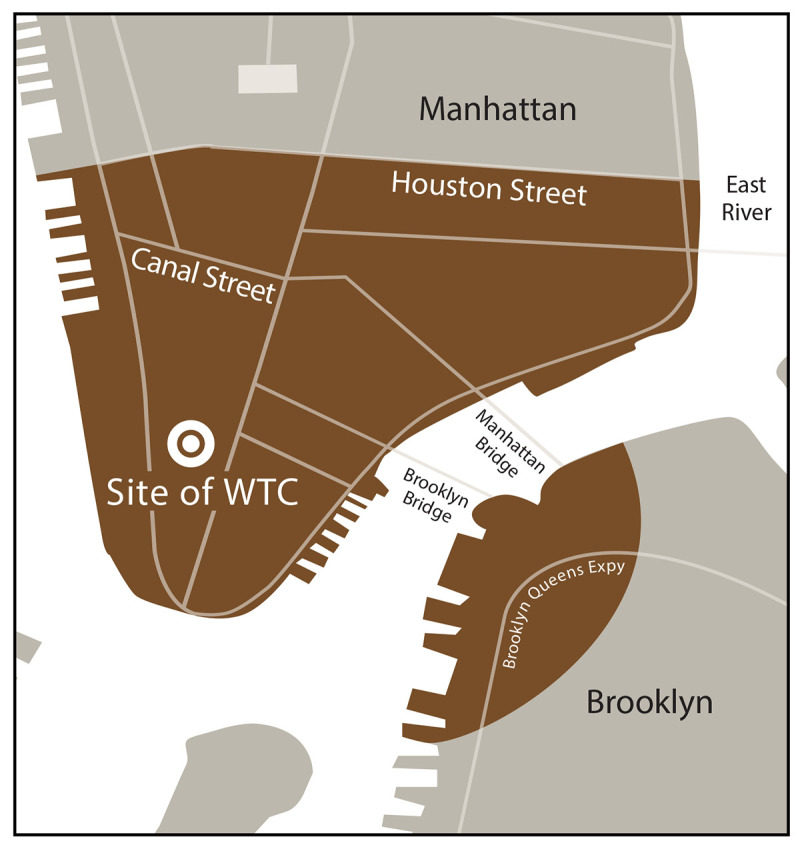
New York City disaster area* used to determine enrollment eligibility for survivors^†^ of the 9/11 attacks — World Trade Center Health Program **Abbreviations:** NYC = New York City; WTC = World Trade Center. * The NYC disaster area for the WTC Health Program is defined as the area in Manhattan south of Houston Street and any block in Brooklyn wholly or partially contained within a 1.5-mile radius of the former WTC complex. ^†^ Survivor is defined as a person who was present in the dust or dust cloud on 9/11 or who worked, lived, or attended school, child care centers, or adult day care centers in the NYC disaster area.
2. Pentagon site in Arlington, Virginia
3. Shanksville, Pennsylvania, site

Qualifying time periods vary substantially by different eligibility groups but generally range from September 11, 2001, through July 31, 2002, with some exceptions for survivor eligibility that extend through May 31, 2003. The hours of exposure used to determine eligibility also vary among groups but generally range from presence in the dust cloud to 4–80 hours of exposure, depending on the location and dates of exposure. Responders and survivors who were enrolled in the federally sponsored predecessor programs (i.e., the Medical Monitoring and Treatment Program, the National Responder Program, and the WTC Environmental Health Center Community Program) before July 1, 2011, were automatically enrolled in the WTC Health Program. More detailed information about eligibility for the WTC Health Program is available (https://www.cdc.gov/wtc/eligiblegroups.html).

Prospective members submit WTC Health Program enrollment application forms and supporting documents to the WTC Health Program for approval (https://www.cdc.gov/wtc/apply.html). WTC Health Program staff members review applications and determine approval status or request additional information. Once approved, applicants are enrolled, and an initial health evaluation is scheduled at a designated Clinical Center of Excellence (CCE) or with a clinical provider in the Nationwide Provider Network (NPN) (Box 2). This initial health evaluation includes medical and exposure histories, a physical examination, and any additional needed medical testing and also might include the following services: 9/11 exposure assessment; medical history and mental health questionnaires; physical examination; spirometry/pulmonary function testing; vital signs; blood tests; chest radiographs, if medically necessary; electrocardiography, if medically necessary; and urinalysis.

BOX 2 **Clinical health care providers for Clinical Centers of Excellence and the Nationwide Provider Network**

**Clinical Centers of Excellence for responders and survivors in the New York metropolitan area**

Responders

- Fire Department of the City of New York
- Icahn School of Medicine at Mount Sinai
- New York University Grossman School of Medicine
- Northwell Health
- Rutgers University
- State University of New York, Stony Brook

Survivors

- NYC Health + Hospital System
- William Street Clinic

**Nationwide Provider Network for responders and survivors outside the New York metropolitan area**

- Nationwide network of clinical providers administered by Logistics Health, Inc.

**Abbreviation:** NYC = New York City.

The initial health evaluation for responders is the baseline monitoring examination, and the responders are eligible for yearly follow-up examinations, which are provided at no cost to the responder. The initial health evaluation for survivors is a no-cost, one-time only examination. For survivors, if no WTC-related health condition is diagnosed at this initial health evaluation, then the survivor member is not eligible for program health benefits coverage at that time. However, survivor members who develop a WTC-related health condition at a later date can contact their designated CCE or NPN to determine whether the condition could be certified by the WTC Health Program. If they eventually receive a WTC-related health condition diagnosis and become certified for that condition, their status changes to certified-eligible survivor, and they are eligible for yearly follow-up examinations as well as other benefits related to the certified WTC-related health condition.

#### Certification

Certification of a WTC-related health condition is based on the requirement that 9/11 exposures were substantially likely to have been a significant factor in aggravating, contributing to, or causing a health condition. A comprehensive list of WTC-related health conditions covered by the WTC Health Program is available (*12*). In addition, the WTC Health Program covers medically associated health conditions, which are conditions that result from the treatment or progression of a certified WTC-related health condition. Certification requests can be made by CCE or NPN clinical providers at the initial health evaluation visit or during any other clinical interactions between members and the clinical providers in the program. These requests are then submitted to designated, specially trained WTC Health Program staff members who review them for approval, denial, or requests for additional information from the CCEs or NPN. This review process follows policies and procedures established by the administrator of the WTC Health Program. Once members become certified with a WTC-related health condition, they are able to receive authorized program health benefits. These authorized program health benefits are provided as clinical services from a CCE or NPN provider and are submitted as claims to the WTC Health Program. More detailed information on WTC Health Program policies and certification criteria is available (https://www.cdc.gov/wtc/policies.html and https://www.cdc.gov/wtc/ppm.html#certification).

### Data Source and Collection

The WTC Health Program uses a multilayer administrative claims system to process members’ authorized program health benefits. This administrative claims system involves multiple entities and levels of claims processing. These entities and levels include CCE and NPN internal and external clinical providers and their billing systems, CCE and NPN institutional billing systems, various third-party clearinghouses or claims administrators, and federal administrative and payer systems. The WTC Health Program claims process consists of internal and external provider claims that are submitted to third-party claims administrators who review claims for basic program and claim requirements. These administrators then send the claims to CCE or NPN providers for an additional review of member information and WTC Health Program codebook compliance, as well as any other necessary program authorizations. A claim might be returned to any of the involved entities for clarification of discrepancies with billing or program policies. If no issues are found with the claim or all discrepancies have been resolved, the claim is approved by the federal administrative and payer system for payment to the program-affiliated clinical providers delivering health services to members.

WTC Health Program administrative claims data are collected, stored, and secured in a database and managed by data contractors on behalf of the WTC Health Program. The administrative claims data are then transferred to the health program evaluation section of the WTC Health Program in quarterly data extracts for various internal data analyses and evaluations. The restricted data (which are not available for public use) presented in this report are derived from the June 2021 quarterly claims data extract and are limited to the 2012–2020 calendar years. Typically, 90% of claims are processed within 6 months from the date of service rendered by the clinical provider (Health Research and Analysis, World Trade Center Health Program Lag Analysis Report: Calendar Years 2012 to 2018, unpublished data, 2019). Because the WTC Health Program began operating on July 1, 2011, administrative claims data for July–December 2011 might affect comparability with later years; therefore, these months were not included in this analysis. Only complete calendar years of administrative claims data were included in the analysis in this report. Administrative claims data used by the WTC Health Program are protected under the Health Insurance Portability and Accountability Act of 1996 (HIPAA), which requires the WTC Health Program to maintain the privacy and security of members’ personal health information. The WTC Health Program is authorized to collect, analyze, and report claims data by Section 3304 of the Zadroga Act (*10*). For the analysis in this report, no identifiable personal health information was needed to construct any of the outcomes. This activity was reviewed by CDC and was conducted consistent with applicable federal law and CDC policy (*13*).

### Definitions and Variables

This report describes selected WTC Health Program indicators among enrolled member responders and survivors for 2012–2020. Enrolled members are defined as persons who have been part of the WTC Health Program at any point in a full calendar year. Members are described by age, sex, deceased status, CCE or NPN provider, and geographic area of residence. Deaths among members are tracked by the WTC Health Program for administrative purposes only; no information on cause of death is collected. Geographic location of members (i.e., U.S. state) is based on the most recent demographic information within a calendar year. Certified members are defined as persons who have at least one WTC-related health condition certified for treatment by the WTC Health Program. Certified health conditions are categorized as either cancer or noncancer conditions, and the distribution of these conditions is ranked by year. Some members were certified both for cancer and noncancer conditions. Cancer certifications were grouped for data analysis using the National Cancer Institute Surveillance, Epidemiology, and End Results program classification (https://seer.cancer.gov/codrecode/1969_d09172004). The WTC Health Program began certifying members for cancer conditions in October 2012; therefore, this report uses 2013 as the base year for cancer certification and data analysis. Members who had an annual examination are defined as persons who received an examination during a specified calendar year based on the date of service (i.e., the baseline monitoring examination for responders, annual monitoring for responders, the initial health evaluation for screening-eligible survivors, or annual monitoring for certified-eligible survivors). A treated member is defined as a member with a paid medical or pharmacy claim within the specified year, excluding paid transactions for any monitoring and screening examinations. In 2018, a newly contracted CCE, the William Street Clinic, began providing monitoring and treatment services for the survivor population.

### Analysis

All statistical analyses were performed using SAS (version 9.4; SAS Institute). Ordinary least-squares linear regression was used to examine temporal trends in select variables during 2012–2020. Two-tailed t-tests were used to calculate p values. A level of α<0.05 was used to determine whether the slope parameter differed significantly from the null. The relative percent difference between temporal endpoints was also calculated: ([n_2020_ − n_2012_] / n_2012_) × 100. In tables, counts <10 are suppressed to maintain confidentiality.

## Results

### Enrollment

**FIGURE 3:**
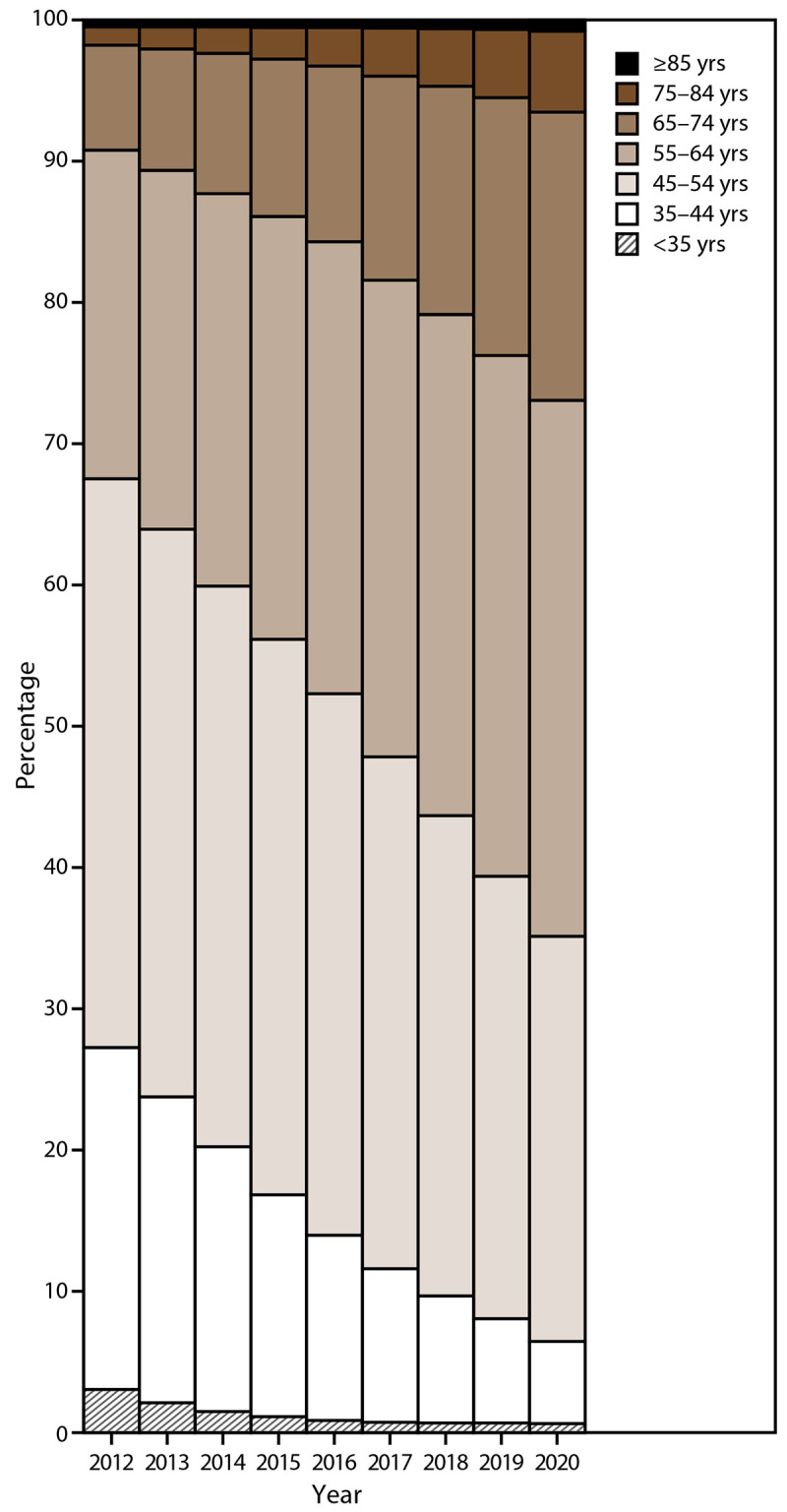
Enrolled members* (responders and survivors),^†^ by age group^§^ and year — World Trade Center Health Program, United States, 2012–2020 * Enrolled member is defined as a person whose application has been approved by the World Trade Center Health Program and is eligible for program health benefits coverage. ^†^ Responder is defined as a person who was involved in rescue, response, recovery, cleanup, and related support activities after the September 11, 2001, terrorist attacks; survivor is defined as a person who was present in the dust or dust cloud on 9/11 or who worked, lived, or attended school, child care centers, or adult day care centers in the New York City disaster area. ^§^ Percentages for members aged ≥85 years were <1% for all years, ranging from 0.2% in 2012 to 0.8% in 2020; percentages for members aged <35 years were <5% for all years, ranging from 3.1% in 2012 to 0.6% in 2020.

**FIGURE 4:**
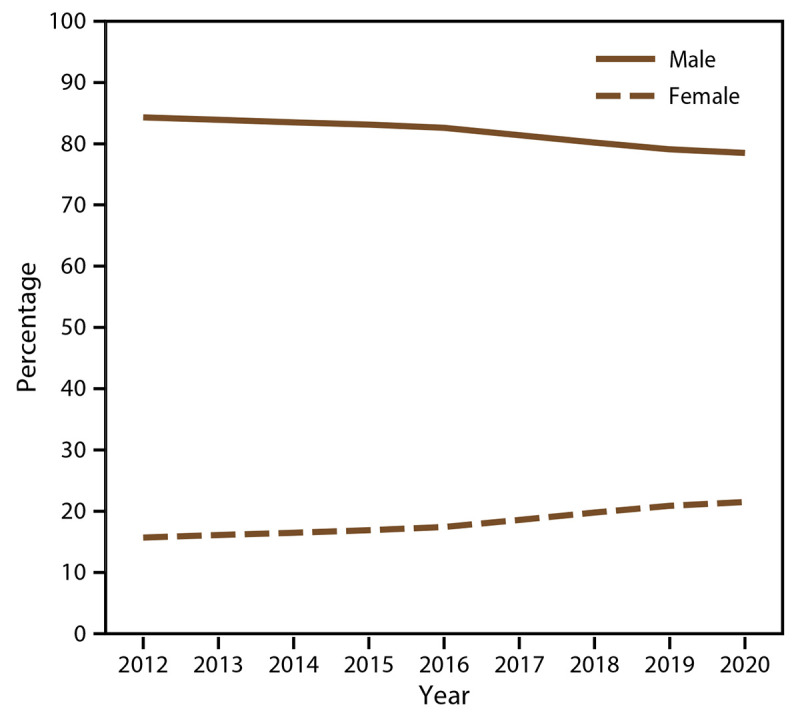
Enrolled members* (responders and survivors),^†^ by sex and year — World Trade Center Health Program, United States, 2012–2020 * Enrolled member is defined as a person whose application has been approved by the World Trade Center Health Program and is eligible for program health benefits coverage. ^†^ Responder is defined as a person who was involved in rescue, response, recovery, cleanup, and related support activities after the September 11, 2001, terrorist attacks; survivor is defined as a person who was present in the dust or dust cloud on 9/11 or who worked, lived, or attended school, child care centers, or adult day care centers in the New York City disaster area.

In 2020, the total number of WTC Health Program enrolled members was 104,223, of which 73.4% (n = 76,543) were responders and 26.6% (n = 27,680) were survivors. From 2012 to 2020, the total number of enrolled WTC Health Program members increased by 67.6% (from 62,171 in 2012 to 104,223 in 2020). The survivor population enrollment rate increased by 364.4% (from 9.6% [n = 5,960] in 2012 to 26.6% [n = 27,680] in 2020) (Table 2). As expected in a closed cohort health program, the age of WTC Health Program members has continued to increase; the median age of members was 51 years (IQR: 44−57 years) in 2012 and 59 years (IQR: 52−66 years) in 2020 (Figure 3). Overall, most WTC Health Program members were male (Figure 4), although recent trends show that the percentage of females in the survivor population is higher than in the responder population (Table 2). The total number of deceased WTC Health Program members increased over time, with the greatest increase occurring among survivors (3,312.5% increase, from eight deceased members in 2012 to 273 deceased members in 2020) (Table 3). In addition, the median age of decedents increased from 60 years (IQR: 52−67 years) in 2012 to 67 years (IQR: 60−75 years) in 2020. The total number of enrolled WTC Health Program members generally increased at the respective CCEs and NPN program, with the greatest increase at the NPN program (310.0% increase, from 5,426 in 2012 to 22,244 in 2020) and the NYC Health + Hospital CCE (136.4% increase, from 5,869 in 2012 to 13,876 in 2020) (Table 4). Thus, in 2020, among enrolled members, 21% (n = 22,244) were enrolled and received care from an NPN provider, and 13% (n = 13,876) received care at the NYC Health + Hospital CCE. Overall, most WTC Health Program members lived in New York (71.7%), New Jersey (9.3%), and Florida (5.7%), although members live in every U.S. state.

**TABLE 2:** Number and percentage of enrolled members* (responders and survivors),^†^ by sex and year — World Trade Center Health Program, United States, 2012–2020

Enrolled members	2012	2013	2014	2015	2016	2017	2018	2019	2020	Relative change, %^§^
	No. (%)	No. (%)	No. (%)	No. (%)	No. (%)	No. (%)	No. (%)	No. (%)	No. (%)	2012 to 2020
**Responder**	**56,211 (90.4)**	**58,279 (89.3)**	**60,816 (88.0)**	**62,479 (87.3)**	**64,197 (86.1)**	**67,187 (82.7)**	**71,279 (79.4)**	**74,530 (75.8)**	**76,543 (73.4)**	**36.2**
Male	49,441 (88.0)	51,298 (88.0)	53,563 (88.1)	54,999 (88.0)	56,465 (88.0)	59,059 (87.9)	62,672 (87.9)	65,504 (87.9)	67,267 (87.9)	36.1
Female	6,770 (12.0)	6,981 (12.0)	7,253 (11.9)	7,480 (12.0)	7,732 (12.0)	8,128 (12.1)	8,607 (12.1)	9,026 (12.1)	9,276 (12.1)	37.0
**Survivor**	**5,960 (9.6)**	**6,988 (10.7)**	**8,258 (12.0)**	**9,124 (12.7)**	**10,364 (13.9)**	**14,035 (17.3)**	**18,543 (20.6)**	**23,803 (24.2)**	**27,680 (26.6)**	**364.4**
Male	2,971 (49.8)	3,483 (49.8)	4,098 (49.6)	4,510 (49.4)	5,129 (49.5)	7,030 (50.1)	9,405 (50.7)	12,316 (51.7)	14,528 (52.5)	389.0
Female	2,989 (50.2)	3,505 (50.2)	4,160 (50.4)	4,614 (50.6)	5,235 (50.5)	7,005 (49.9)	9,138 (49.3)	11,487 (48.3)	13,152 (47.5)	340.0
**Total no.**	**62,171**	**65,267**	**69,074**	**71,603**	**74,561**	**81,222**	**89,822**	**98,333**	**104,223**	**67.6**

* Enrolled member is defined as a person whose application has been approved by the World Trade Center Health Program and is eligible for program health benefits coverage.

^†^ Responder is defined as a person who was involved in rescue, response, recovery, cleanup, and related support activities after the September 11, 2001, terrorist attacks; survivor is defined as a person who was present in the dust or dust cloud on 9/11 or who worked, lived, or attended school, child care centers, or adult day care centers in the New York City disaster area.

^§^ Ordinary least-squares linear regression, with p value from two-tailed t-test. All values are statistically significant (p<0.01).

**TABLE 3:** Number, percentage, and median age of deceased members* (responders and survivors),^†^by year — World Trade Center Health Program, United States, 2012–2020

Members	2012	2013	2014	2015	2016	2017	2018	2019	2020	Relative change, %^§^
	No. (%)	No. (%)	No. (%)	No. (%)	No. (%)	No. (%)	No. (%)	No. (%)	No. (%)	2012 to 2020
Responder	174 (95.6)	195 (92.0)	229 (76.3)	269 (86.5)	298 (83.9)	339 (81.3)	422 (73.3)	373 (68.8)	448 (62.1)	157.5
Survivor	8 (4.4)	17 (8.0)	71 (23.7)	42 (13.5)	57 (16.1)	78 (18.7)	154 (26.7)	169 (31.2)	273 (37.9)	3,312.5
**Median age, yrs (IQR)**	60.1 (51.8−67.1)	62.5 (53.5−69.8)	62.8 (55.4−69.5)	63.6 (55.7−72.5)	63.7 (55.6−72.5)	64.0 (55.8−72.0)	65.9 (58.1−73.5)	66.7 (59.9−74.6)	67.0 (59.6−74.7)	NA
**Total no.**	**182**	**212**	**300**	**311**	**355**	**417**	**576**	**542**	**721**	**296.2**

**Abbreviations:** IQR = interquartile range; NA = not applicable.

* Deaths among members are tracked by the World Trade Center Health Program for administrative purposes only; no information on cause of death is collected.

^†^ Responder is defined as a person who was involved in rescue, response, recovery, cleanup, and related support activities after the September 11, 2001, terrorist attacks; survivor is defined as a person who was present in the dust or dust cloud on 9/11 or who worked, lived, or attended school, child care centers, or adult day care centers in the New York City disaster area.

^§^ Ordinary least-squares linear regression, with p value from two-tailed t-test. All values are statistically significant (p<0.01).

**TABLE 4:** Number and percentage of enrolled members* (responders and survivors),^†^ by health care provider and year — World Trade Center Health Program, United States, 2012–2020

Health care provider	2012	2013	2014	2015	2016	2017	2018	2019	2020	Relative change, %^§^
	No. (%)	No. (%)	No. (%)	No. (%)	No. (%)	No. (%)	No. (%)	No. (%)	No. (%)	2012 to 2020
**Clinical Centers of Excellence (responders in the NY metropolitan area)**										
FDNY	15,841 (25.5)	15,774 (24.2)	15,699 (22.7)	15,621 (21.8)	15,549 (20.9)	15,459 (19.0)	15,375 (17.1)	15,237 (15.5)	15,143 (14.5)	−4.4
Icahn School of Medicine at Mount Sinai	20,763 (33.4)	21,138 (32.4)	21,696 (31.4)	22,016 (30.7)	22,165 (29.7)	22,780 (28.0)	23,698 (26.4)	24,067 (24.5)	24,326 (23.3)	17.2
NYU Grossman School of Medicine	2,103 (3.4)	2,160 (3.3)	2,294 (3.3)	2,343 (3.3)	2,384 (3.2)	2,510 (3.1)	2,628 (2.9)	2,740 (2.8)	2,813 (2.7)	33.8
Northwell Health	2,910 (4.7)	2,995 (4.6)	3,090 (4.5)	3,193 (4.5)	3,335 (4.5)	3,590 (4.4)	3,902 (4.3)	4,098 (4.2)	4,209 (4.0)	44.6
Rutgers University	2,335 (3.8)	2,418 (3.7)	2,559 (3.7)	2,663 (3.7)	2,812 (3.8)	3,133 (3.9)	3,607 (4.0)	4,097 (4.2)	4,419 (4.2)	89.3
SUNY, Stony Brook	6,923 (11.1)	7,712 (11.8)	8,202 (11.9)	8,564 (12.0)	9,019 (12.1)	9,817 (12.1)	10,883 (12.1)	11,751 (12.0)	12,283 (11.8)	77.4
**Clinical Centers of Excellence (survivors in the NY metropolitan area)**										
NYC Health + Hospitals	5,869 (9.4)	6,711 (10.3)	7,717 (11.2)	8,355 (11.7)	9,289 (12.5)	11,637 (14.3)	11,250 (12.5)	12,653 (12.9)	13,876 (13.3)	136.4
William Street Clinic	—^¶^	—^¶^	—^¶^	—^¶^	—^¶^	—^¶^	2,829 (3.1)	4,178 (4.2)	4,910 (4.7)	—**
**Nationwide Provider Network (responders and survivors outside the NY metropolitan area)**										
Health providers nationwide	5,426 (8.7)	6,358 (9.7)	7,816 (11.3)	8,847 (12.4)	10,007 (13.4)	12,295 (15.1)	15,650 (17.4)	19,512 (19.8)	22,244 (21.3)	310.0

**Abbreviations:** FDNY = Fire Department of the City of New York; NY = New York; NYC = New York City; NYU = New York University; SUNY = State University of New York.

* Enrolled member is defined as a person whose application has been approved by the World Trade Center Health Program and is eligible for program health benefits coverage.

^†^ Responder is defined as a person who was involved in rescue, response, recovery, cleanup, and related support activities after the September 11, 2001, terrorist attacks; survivor is defined as a person who was present in the dust or dust cloud on 9/11 or who worked, lived, or attended school, child care centers, or adult day care centers in the New York City disaster area.

^§^ Ordinary least-squares linear regression, with p value from two-tailed t-test. All values are statistically significant (p<0.01).

^¶^ Data not available.

** Not calculated.

### Certification

**FIGURE 5:**
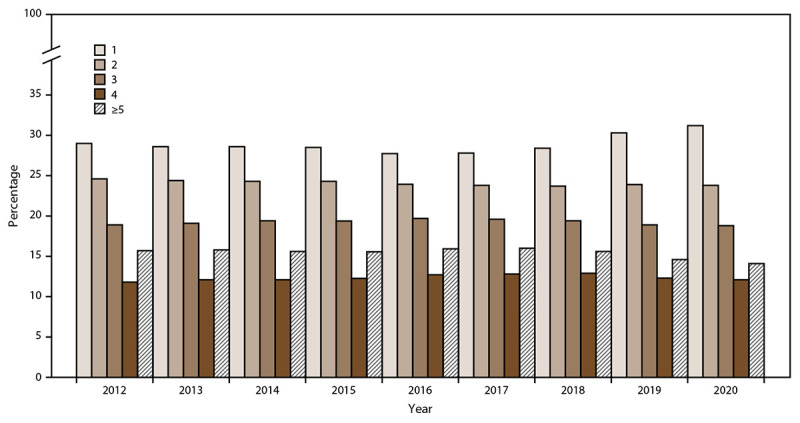
Percentage of certified members* (responders and survivors)^†^ with health condition certifications,^§^ by number of certifications per member and year — World Trade Center Health Program, United States, 2012–2020 * Certified member is defined as a person who has been enrolled in the World Trade Center Health Program, has received their initial health evaluation or baseline monitoring examination, and has been determined to have a program covered condition via a review process. ^†^ Responder is defined as a person who was involved in rescue, response, recovery, cleanup, and related support activities after the September 11, 2001, terrorist attacks; survivor is defined as a person who was present in the dust or dust cloud on 9/11 or who worked, lived, or attended school, child care centers, or adult day care centers in the New York City disaster area. ^§^ Certification of a World Trade Center–related health condition is based on the requirement that 9/11 exposures were substantially likely to have been a significant factor in aggravating, contributing to, or causing a health condition.

In 2020, among all enrolled members, 58% (n = 60,433) had at least one certified condition, of which 72.0% (n = 43,503) were responders and 28.0% (n = 16,930) were survivors. The survivor population certification rate increased by 325.8% (from 3,976 in 2012 to 16,930 in 2020) (Table 5). In general, the total number of health condition certifications has increased; the increase was greatest among the survivor population (from 8,889 in 2012 to 35,769 in 2020) (Table 6). Many members have more than one WTC-related health condition certified, with a range of one to five or more (Figure 5); the mean number of certifications per certified member is 2.7.

**TABLE 5:** Number and percentage of certified members* (responders and survivors),^†^ by year — World Trade Center Health Program, United States, 2012–2020

Member	2012	2013	2014	2015	2016	2017	2018	2019	2020	Relative change, %^§^
	No. (%)	No. (%)	No. (%)	No. (%)	No. (%)	No. (%)	No. (%)	No. (%)	No. (%)	2012 to 2020
Responder	24,217 (85.9)	26,049 (85.4)	28,061 (84.7)	29,921 (84.1)	31,893 (83.5)	34,251 (82.4)	37,574 (80.6)	40,829 (74.5)	43,503 (72.0)	79.6
Survivor	3,976 (14.1)	4,465 (14.6)	5,086 (15.3)	5,661 (15.9)	6,308 (16.5)	7,300 (17.6)	9,069 (19.4)	13,945 (25.5)	16,930 (28.0)	325.8
**Total no.**	**28,193**	**30,514**	**33,147**	**35,582**	**38,201**	**41,551**	**46,643**	**54,774**	**60,433**	**114.4**

* Certified member is defined as a person who has been enrolled in the World Trade Center Health Program, has received their initial health evaluation or baseline monitoring examination, and has been determined to have a program covered condition via a review process.

^†^ Responder is defined as a person who was involved in rescue, response, recovery, cleanup, and related support activities after the September 11, 2001, terrorist attacks; survivor is defined as a person who was present in the dust or dust cloud on 9/11 or who worked, lived, or attended school, child care centers, or adult day care centers in the New York City disaster area.

**^§^** Ordinary least-squares linear regression, with p value from two-tailed t-test. All values are statistically significant (p<0.01).

**TABLE 6:** Number and percentage of health condition certifications* among members, by member type (responders and survivors)^†^ and year — World Trade Center Health Program, United States, 2012–2020

Member	2012	2013	2014	2015	2016	2017	2018	2019	2020	Relative change, %^§^
	No. (%)	No. (%)	No. (%)	No. (%)	No. (%)	No. (%)	No. (%)	No. (%)	No. (%)	2012 to 2020
Responder	69,972 (88.7)	75,702 (88.3)	81,367 (87.6)	86,875 (87.1)	93,904 (86.6)	101,206 (85.8)	110,682 (84.4)	119,587 (80.0)	127,061 (78.0)	81.6
Survivor	8,889 (11.3)	10,053 (11.7)	11,548 (12.4)	12,912(12.9)	14,481 (13.4)	16,708 (14.2)	20,430 (15.6)	29,884 (20.0)	35,769 (22.0)	302.4
**Total no.**	**78,861**	**85,755**	**92,915**	**99,787**	**108,385**	**117,914**	**131,112**	**149,471**	**162,830**	**106.5**

* Certification of a World Trade Center–related health condition is based on the requirement that 9/11 exposures were substantially likely to have been a significant factor in aggravating, contributing to, or causing a health condition. A comprehensive list of World Trade Center–related health conditions covered by the World Trade Center Health Program is available (https://www.cdc.gov/wtc/handbook.html#certifications).

^†^ Responder is defined as a person who was involved in rescue, response, recovery, cleanup, and related support activities after the September 11, 2001, terrorist attacks; survivor is defined as a person who was present in the dust or dust cloud on 9/11 or who worked, lived, or attended school, child care centers, or adult day care centers in the New York City disaster area.

**^§^** Ordinary least-squares linear regression, with p value from two-tailed t-test. All values are statistically significant (p<0.01).

In 2020, the total numbers of cancer and noncancer certifications among members were 20,612 and 50,611, respectively. Both cancer and noncancer certification rates increased, with the greatest increase among survivors (4,150.5% increase in cancer certifications, from 188 in 2013 to 7,991 in 2020; 207.8% increase in noncancer certifications, from 3,971 in 2012 to 12,222 in 2020) (Table 7). In 2013, among certified survivor members, 0.6% (n = 188) had a cancer certification, and in 2012, 14% (n = 3,971) had a noncancer certification. Thus, in 2020, among certified survivor members, 13% (n = 7,991) had a cancer certification and 20% (n = 12,222) had a noncancer certification. In 2020, the top five certified cancer conditions were 1) skin cancer (30.7% [n = 6,334]), 2) male genital system cancers (23.3% [n = 4,795]), 3) in situ neoplasms (11.5% [n = 2,366]), 4) breast cancer (8.4% [n = 1,730]), and 5) digestive system cancers (7.8% [n = 1,603]). In addition, the top five noncancer certified conditions were 1) upper respiratory disease (64.0% [n = 32,404]), 2) gastroesophageal reflux disorder (56.4% [n = 28,553]), 3) obstructive airway disease (49.1% [n = 24,839]), 4) posttraumatic stress disorder (PTSD) (23.1% [n = 11,699]), and 5) depression (11.9% [n = 6,006]). Overall, in 2020, the total number of noncancer-certified members (n = 50,611) were grouped into three major noncancer certified categories: 1) aerodigestive, which includes obstructive airway disease, upper respiratory disease, gastroesophageal reflux disorder, and interstitial lung disease (91.0% [n = 46,072]); 2) mental health, which includes PTSD, depression, anxiety, adjustment disorder, and substance abuse (36.5% [n = 18,450]); and 3) musculoskeletal and acute traumatic injuries (2.7% [n = 1,358]), which includes spine, extremity, and head trauma. (Members could have one or more certifications in each group; thus, percentages do not add up to 100%.)

**TABLE 7:** Number and percentage of cancer and noncancer certifications* among members, by member type (responders and survivors)^†^ — World Trade Center Health Program, United States, 2012–2020

Certification and member	2012	2013	2014	2015	2016	2017	2018	2019	2020	Relative change, %^§^
	No. (%)	No. (%)	No. (%)	No. (%)	No. (%)	No. (%)	No. (%)	No. (%)	No. (%)	2012 to 2020^¶^
**Cancer**										
Responder	NA	1,682 (89.9)	2,864 (87.3)	4,057 (87.0)	5,151 (85.3)	6,671 (81.7)	8,637 (76.5)	10,744 (65.1)	12,621 (61.2)	650.4
Survivor	NA	188 (10.1)	416 (12.7)	605 (13.0)	890 (14.7)	1,497 (18.3)	2,652 (23.5)	5,753 (34.9)	7,991 (38.8)	4,150.5**
**Total no.**	NA	**1,870**	**3,280**	**4,662**	**6,041**	**8,168**	**11,289**	**16,497**	**20,612**	**1,002.3**
**Noncancer**										
Responder	24,155 (85.9)	25,460 (85.3)	27,030 (84.6)	28,407 (84.0)	30,038 (83.6)	31,795 (82.9)	34,233 (81.7)	36,560 (77.4)	38,389 (75.9)	58.9
Survivor	3,971 (14.1)	4,388 (14.7)	4,904 (15.4)	5,394 (16.0)	5,902 (16.4)	6,566 (17.1)	7,670 (18.3)	10,688 (22.6)	12,222 (24.1)	207.8
**Total no.**	**28,126**	**29,848**	**31,934**	**33,801**	**35,940**	**38,361**	**41,903**	**47,248**	**50,611**	**79.9**

**Abbreviation:** NA = not applicable.

* Certifications of a World Trade Center–related health condition is based on the requirement that 9/11 exposures were substantially likely to have been a significant factor in aggravating, contributing to, or causing a health condition. A comprehensive list of World Trade Center-related health conditions covered by the World Trade Center Health Program is available at https://www.cdc.gov/wtc/handbook.html#certifications

^†^ Responder is defined as a person who was involved in rescue, response, recovery, cleanup, and related support activities after the September 11, 2001, terrorist attacks; survivor is defined as a person who was present in the dust or dust cloud on 9/11 or who worked, lived, or attended school, child care centers, or adult day care centers in the New York City disaster area.

^§^ Ordinary least-squares linear regression, with p value from two-tailed t-test. All values are statistically significant (p<0.01).

^¶^ The World Trade Center Health Program began certifying members for cancer conditions in October 2012; therefore, the percent relative change difference for cancer certifications is calculated from 2013 to 2020.

** p = 0.003.

### Monitoring and Treatment

**FIGURE 6:**
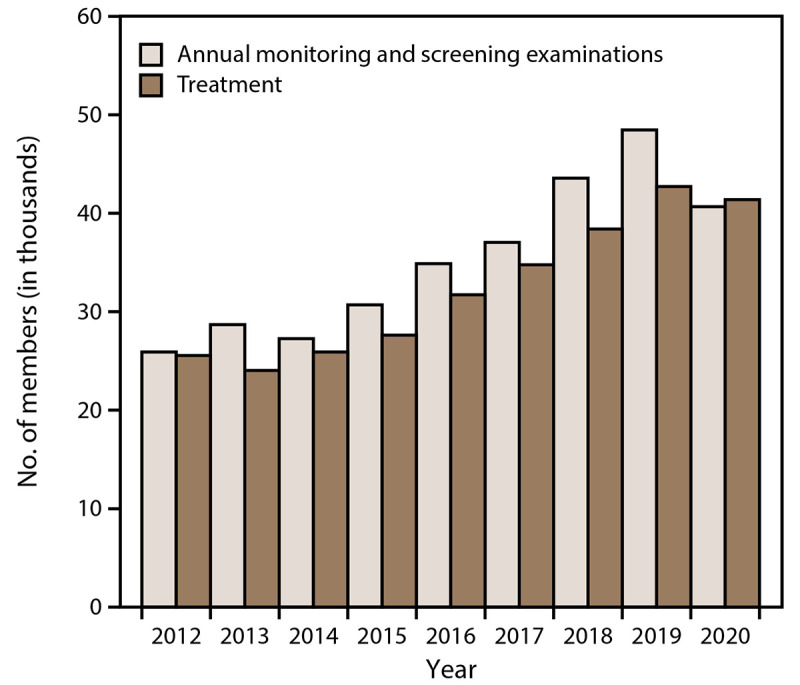
Number of members (responders and survivors)* who received annual monitoring and screening examinations^†^ and treatment,^§^ by year — World Trade Center Health Program, United States, 2012–2020 * Responder is defined as a person who was involved in rescue, response, recovery, cleanup, and related support activities after the September 11, 2001, terrorist attacks; survivor is defined as a person who was present in the dust or dust cloud on 9/11 or who worked, lived, or attended school, child care centers, or adult day care centers in the New York City disaster area. ^†^ Members who received an annual examination are defined as persons who received an examination (i.e., the baseline monitoring examination for responders, annual monitoring for responders, the initial health evaluation for screening-eligible survivors, or annual monitoring for certified-eligible survivors) during a full calendar year based on the date of service. ^§^ Treated member is defined as any member who had a paid medical or pharmacy claim within the specified calendar year. Members (responders and survivors) must be certified to receive treatment.

In 2020, the total number of WTC Health Program members who had annual monitoring and screening examinations was 40,666 (Figure 6). An average of 35,245 annual monitoring and screening examinations occurred per calendar year during 2012–2020. From 2012 to 2020, the total number of members who had annual monitoring and screening examinations increased by 56.9% (from 25,918 in 2012 to 40,666 in 2020). In 2020, the total number of members who received treatment was 41,387. The average number of members receiving treatment per calendar year was 32,458. From 2012 to 2020, the total number of members who received treatment increased by 62.0% (from 25,553 in 2012 to 41,387 in 2020).

### Research

**FIGURE 7:**
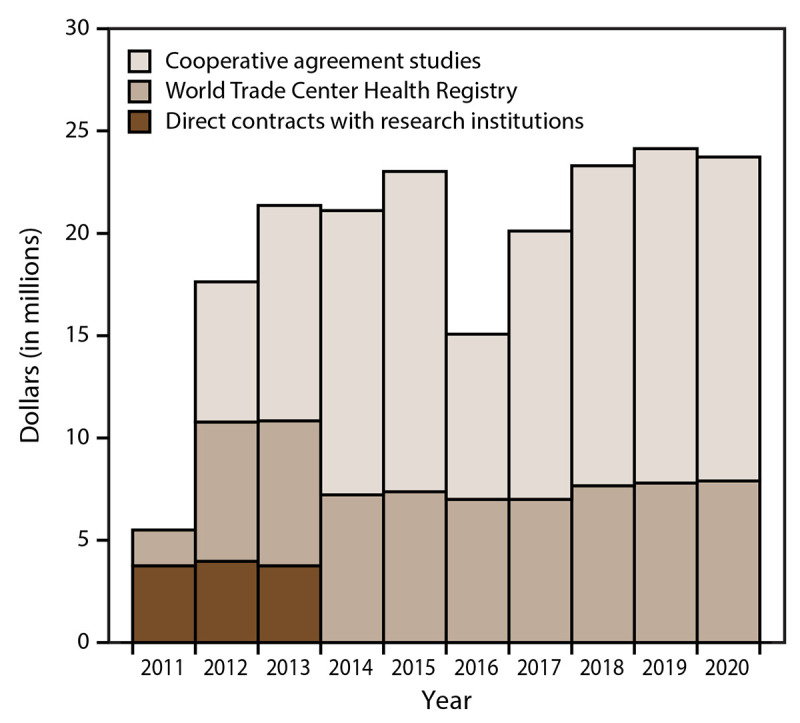
Research funding, by dollar amount (in millions), funding type or program, and year awarded — World Trade Center Health Program, United States, 2011–2020

During 2011–2020, the WTC Health Program awarded a total of $195 million in research funding through cooperative research agreements ($115.9 million), contracts ($11.5 million), and the WTC Health Registry ($67.6 million) (Figure 7). As of 2020, a total of 321 peer-reviewed publications had been funded by the WTC Health Program.

## Discussion

This report is the first to provide a comprehensive summary of selected indicators of the WTC Health Program for 2012–2020 and includes several important findings. First, as of December 31, 2020, approximately 104,000 persons were enrolled in the WTC Health Program. Most members are male, with more females among survivors, and most members live in the New York and New Jersey area. Although responders comprised the highest proportion of enrolled members, survivors accounted for the greatest increase in the rate of enrollment. Second, approximately 60,000 members had been certified with at least one WTC-related health condition. Although responders had the highest proportion of certified members, survivors accounted for the greatest percentage change of certified members. In general, WTC Health Program members have a mean of 2.7 certifications per certified member, indicating a propensity for comorbid conditions. In addition, although the numbers of both cancer and noncancer certifications among members have increased, the survivor population accounted for the greatest percentage change. Skin cancer, male genital system cancers, and in situ neoplasms (e.g., skin or breast) are the most commonly certified cancer conditions. For noncancer conditions, WTC Health Program members are primarily certified for conditions in the aerodigestive and mental health categories. Third, in 2020, a total of 40,666 WTC Health Program members received annual monitoring and screening examinations, and 41,387 WTC Health Program members received treatment. During 2012–2020, the total number of annual monitoring and screening examinations and treatments increased. In 2020, however, 7,790 fewer annual monitoring and screening examinations were completed and 1,333 fewer treatments were received than in 2019. This absolute difference in numbers might have been a result of the COVID-19 pandemic, which caused widespread facility closures and increased use of telemedicine and other programmatic measures. Furthermore, submission of administrative claims might have been delayed due to clinical and administrative adjustments during the pandemic. Therefore, additional investigations might explain this finding.

Since the inception of the WTC Health Program, its members have predominantly been middle-aged (median age of 51 years) and male, which has aligned with the higher proportion of responders compared with survivors. As the members age, health service use and costs within the WTC Health Program are expected to increase because chronic diseases, comorbidities, and other health-related conditions unrelated to WTC exposures are more common in older populations, which might complicate the clinical management of WTC-related health conditions. The data also suggest that WTC Health Program members at CCEs and the NPN program dedicated to serving survivors have experienced a moderate increase in enrolled members. Noting the increasing survivor enrollment, the WTC Health Program established an additional clinic in 2018 to manage the increasing demand for program services. Although the WTC Health Program has been primarily based in the New York and New Jersey area, there have been increasing numbers of members in neighboring states and more southern states (e.g., Pennsylvania, Virginia, Florida, and North Carolina), which is expanding the location of NPN care. Continued monitoring of these types of data is warranted to identify emerging trends among members, contributing factors to these trends, and other possible programmatic implications.

Since 2012, the number of certifications among members has increased both for cancer and noncancer health conditions. Cancer certifications have increased since the WTC Health Program started certifying this condition in 2012. Existing WTC-related research has found a modestly increased cancer risk in the WTC population, particularly for malignant melanoma, thyroid cancer, and prostate cancer (*14*–*16*). However, existing observational cancer studies have numerous limitations that increase the uncertainty of the findings (*16*,*17*). The WTC Health Program continues to provide health care for members with certified conditions as mandated by the Zadroga Act. Regarding noncancer conditions (i.e., aerodigestive and mental health certification categories), WTC-related studies found that substantial respiratory effects appeared soon after the terrorist attacks, referred to as a WTC cough (*18*–*23*). In addition, a study reported that among persons with a WTC cough, approximately 80% also reported upper airway symptoms (i.e., nasal congestion, nasal drip, and sore throat), and nearly 90% reported symptoms of gastroesophageal reflux disorder (*19*). Overall, studies show higher aerodigestive disorder prevalence among responders compared with the general population (*24*). Similar lower and upper airway symptoms have also been found among survivors (*25*,*26*). In terms of mental health, WTC-related research has found that PTSD, depression, and anxiety disorders are prevalent both among responders and survivors (*27*–*30*). More detailed information about cancer and noncancer WTC-related conditions research is available elsewhere (*16*,*17*,*23*). Continued WTC research has led to better understanding of 9/11 exposures and physical and mental health associations for its members. The research component of the WTC Health Program has guided programmatic decisions on certifying new WTC-related health conditions. Continued research is essential to better understand long-term effects from 9/11 exposures and the treatment of these WTC-related conditions in an aging population.

Although recent WTC Health Program data suggest a slight decrease in the 2020 absolute number of annual monitoring and screening examinations (7,790 fewer than in 2019), the decrease might be a result of the COVID-19 pandemic during 2020. National data show that the number of hospital visits was lower than usual and medical care was delayed or avoided, perhaps because of strict public health mitigation measures or individual concerns related to COVID-19 illness (*31*,*32*). In addition, recent studies showed that NYC was one of the major U.S. cities that was substantially affected early in the COVID-19 pandemic (*33*–*35*). Although the 2020 absolute number of WTC Health Program members receiving treatment was slightly lower than in previous years (1,333 fewer than in 2019), essential services (medical and pharmacy) were generally not discontinued because of the COVID-19 pandemic. Program adjustments, including a rapid transition to telemedicine and care management support, increases in the supply of retail medications, and greater use of the mail-order pharmacy option, facilitated continuity of medically necessary care. Therefore, additional research and surveillance data analyses are warranted to understand the ongoing effects and long-term impact of the COVID-19 pandemic on the WTC Health Program and its members.

As of 2020, the WTC Health Program had funded 321 of approximately 1,000 peer-reviewed publications on 9/11-related health effects. Immediately after the 9/11 attacks and before the Zadroga Act became effective in 2011, WTC research was primarily funded and led by local government and private institutions (i.e., philanthropy). Federal funding also was dedicated to WTC research efforts (i.e., monitoring and screening surveillance and the WTC Health Registry) (*7*). When the Zadroga Act became effective in 2011, the WTC Health Program became the coordinator of and the main funding source for WTC research. This report describes the WTC research funding during 2011–2020, which has been stable since enactment of the Zadroga Act, ensuring the continuity of WTC research projects. A previous study showed that the WTC Health Program research during 2011–2020 has primarily focused on characterizing the etiology and effects of WTC-related conditions (*16*). This first decade (2011–2020) of WTC Health Program research includes research on aerodigestive disorders, adult mental health, cancer, and emerging conditions. More detailed information about the WTC Health Program research projects and agendas and their impact during this first decade have been described (*16*,*36*). WTC research remains a core component of the WTC Health Program because it guides many programmatic decisions, as well as guides CCE and NPN health care providers, stakeholders, and members.

## Public Health Implications

### Public Health and Clinical Care Integration

The September 11, 2001, terrorist attacks marked a new era for public health preparedness and response in the United States. During the first decade (2001–2011) after the terrorist attacks, public health and clinical care activities were conducted primarily by local officials through a federal grant program. These activities were focused on persons who were part of the rescue, recovery, and cleanup efforts (responders) or who were present in the dust or dust cloud on 9/11 or who worked, lived, or attended school, child care centers, or adult day care centers in the NYC disaster area (survivors). In 2011, the Zadroga Act directed the federal government to continue monitoring and providing clinical services to the responders and survivors. In addition, the Zadroga Act expanded coverage to responders of the airplane crashes at the Pentagon and in Shanksville, Pennsylvania, as well as to survivors of the NYC attacks who lived outside of the New York metropolitan area. The Zadroga Act also established the WTC Health Program, which organized and integrated the existing community and local efforts into a coordinated and targeted health benefits program. The Zadroga Act helped implement postdisaster public health activities, including research and coordinated appropriate clinical care interventions. During the short transitional implementation period (6 months) mandated by the Zadroga Act, actions were taken to support continuity of medical care for those in the predecessor programs. Recent WTC Health Program administrative claims data can be used to track trends in enrollment, certification, monitoring and screening, and treatment and guide research components of the program. These data show some increases over time for these program components. Administrative claims and research data have provided vital information for WTC Health Program administrative, clinical, and research decision-making. As such, the initial and ongoing public health activities established the foundations for a focused health benefit program that continues to evolve and adapt to the needs of its members.

## Limitations

The findings in this report are subject to at least seven limitations. First, due to the nature of claims processing, which results in a lag, the number of claims in the 2020 administrative claims data set could be underestimated. This lag might have been augmented by the COVID-19 pandemic during 2020. Second, these analyses did not consider WTC Health Program policy changes that occurred during the study period. However, future analyses can focus on the various WTC Health Program policy changes (i.e., health benefits expansion or coverage). Third, because these analyses are limited to the WTC Health Program population (responders and survivors), results should not be extrapolated to the general population or any other health care program. Fourth, the WTC Health Program administrative claims data set is collected primarily for administrative and surveillance purposes; therefore, clinical outcomes, comorbidities among members, or other clinical factors are unknown. Race and ethnicity data are not included in this administrative claims data set. Nonetheless, independent WTC Health Program research could explore possible linkages between administrative data sets and clinical data sets. In addition, the number of WTC-related certified conditions might be underestimated because of differences in certification practices and reporting by CCEs and the NPN program. Fifth, per the Zadroga Act, care for survivors must be billed to their primary insurer first; the WTC Health Program is considered the secondary payer for care provided for WTC-related conditions. Therefore, treatment reimbursed by the WTC Health Program is not an accurate representation of treatment type or treatment cost for the survivor population. Sixth, because the first-listed diagnosis code on a claim is not necessarily the primary diagnosis code, the interpretation of the top five WTC Health Program certified conditions might not be accurately represented. For example, this use of claims data to characterize certifications might differ from other program reports and statistics that use other data sources of certification data. Finally, caution should be used when comparing these results with other documents, research, and websites that are based on different methods, dates of services, and data sets.

## Future Directions

### Data Systems Interoperability Improvements

WTC Health Program administrative claims data provide programmatic indicators for this limited health benefit plan. After the Zadroga Act was enacted, the goal of setting up the WTC Health Program, including coordinating different health data systems, was achieved. Nonetheless, this multifaceted, interactive and interconnected data system poses a unique challenge for integration into a larger, complex U.S. health care data system. The WTC Health Program health informatics infrastructure could be strengthened into a more timely, accurate, and consistent claims data system. These improvements will need to be integrated with other disparate health data systems (e.g., CCEs and NPN, third-party claims administrators, internal and external clinical providers, and federal payers’ systems) and the complex U.S. health care system. The likely expansion of the WTC Health Program membership into the NPN program will present additional challenges for the interoperability of these data systems. Benefits of strengthening WTC Health Program data systems in this way could 1) increase timeliness of oversight of ongoing services, case management, and plans of care for members through electronic health records; 2) expedite authorization processes; 3) provide timely surveillance data during a local (e.g., a natural disaster such as a hurricane) or a national health emergency response (e.g., COVID-19); 4) enhance quality assurance processes for oversight and detection of potential fraud, waste, and abuse, as mandated by the Zadroga Act; and 5) increase the quality of clinical care for members.

### Research Integration

WTC Health Program members have benefitted from the research conducted over the past 20 years and that has been mandated since 2011 (*16*,*36*). WTC Health Program research provides information on disease etiology and health impact that is needed to guide program decision-making and improve health care (*7*,*16*,*36*). The findings in this report highlight the need for continued research efforts because of persistent and emerging WTC-related health conditions in an aging population. More research is needed on holistic care approaches, such as lifestyle and integrative health interventions. Research into novel treatment methods that are becoming more available to program members is needed. For example, telemedicine became an essential tool for maintaining continuity of care for WTC Health Program members during the COVID-19 pandemic. Research evaluating the efficacy of telemedicine for certain WTC-related health conditions (e.g., mental health) and its effects on other programmatic components (e.g., service use and medication adherence) is needed. Finally, continued community engagement in the research agenda is necessary. For example, WTC Health Program members and researchers have shared program experiences during community events such as the Research to Care Community Engagement in 2017 (https://wwwn.cdc.gov/ResearchGateway/R2C). The integration of surveillance, research, and community engagement can guide the future WTC research agenda.

## Conclusion

After the September 11, 2001, terrorist attacks, a new era of health protection and preparedness began in the United States. The Zadroga Act of 2010 created and established the WTC Health Program, a limited health benefit plan that provides continuous monitoring, screening, and treatment to eligible and enrolled members affected by the attacks. As of 2020, approximately 104,000 responders and survivors had enrolled in the WTC Health Program. On average each year, approximately 35,000 members enrolled in the WTC Health Program received annual monitoring and screening examinations. In addition, on average each year, approximately 32,000 members enrolled in the WTC Health Program received annual treatment. Program data show that all members are aging and the number of survivors is increasing. Data from WTC-related research and the WTC Health Registry have helped guide many programmatic decisions. Timely and regular analysis of integrated administrative and research data can be useful for the overall monitoring, planning, implementation, and evaluation of the WTC Health Program, which will enable the WTC Health Program to provide quality health care services for its enrolled members. Data interoperability and research integration will be important to achieve these and other future program objectives. As the WTC Health Program members age and their diversity increases, new challenges will emerge, and the program will need to adapt to these challenges and the constantly evolving health care needs of its members.
